# The relevance of clinical-pathological correlation in the diagnosis of Cutaneous Rosai-Dorfman disease^☆^

**DOI:** 10.1016/j.abd.2025.501180

**Published:** 2025-08-13

**Authors:** Carolina Ferreira Baldecerra, Patrícia Imai Zanardi, Rafaella Cristine Zanatta, Silvio Cesar Franco Giovanni Filho, Cássio Rafael Moreira

**Affiliations:** Dermatology Outpatient Clinic, Autarquia Municipal de Saúde de Apucarana, Apucarana, PR, Brazil

Dear Editor,

This report describes the case of an 82-year-old woman who sought dermatological evaluation due to two progressively enlarging lesions on her upper back for three months. She reported no symptoms such as fever, sweating, or weight loss. Physical examination revealed two violaceous papular plaques on her upper back, surrounded by similar-looking papules. Dermoscopy revealed a pink background with white structures and telangiectasias (Fig. 1). No adenomegaly was observed on palpation of the main lymph node chains. Complete blood count, biochemical parameters, chest CT scan, and abdominal ultrasound were normal. Histopathological examination revealed a dermal lesion with lymphohistiocytic infiltrate and emperipolesis—i.e., phagocytosis of lymphocytes, plasma cells, and intact red blood cells by histiocytes with ample cytoplasm and conspicuous nucleoli (Fig. 2). Immunohistochemistry was positive in the histiocytes for the markers CD68 (clone PG-M1), Cyclin D1 (clone EP12), and S100 (clone 4C4.9), and negative for CD1a (clone 010) (Fig. 3). Therefore, considering the clinical, laboratory, and histopathological findings together, the diagnosis of Cutaneous Rosai-Dorfman Disease (CRDD) was achieved.

**Figure 1:**
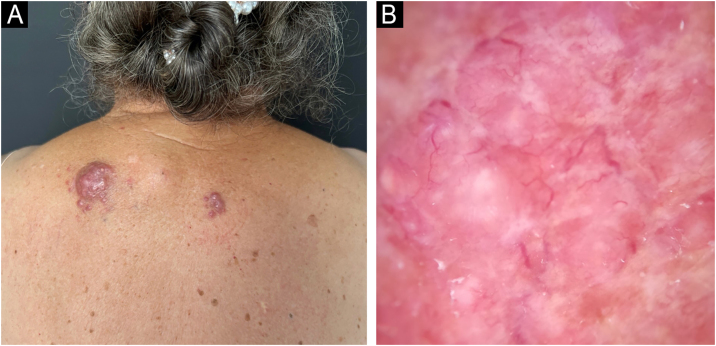
Clinical and dermoscopic photographs of the lesions. (A) Two violaceous papular plaques on the upper back, with surrounding papules of the same color. (B) White structures and telangiectasias with a pink background on dermoscopy.

**Figure 2:**
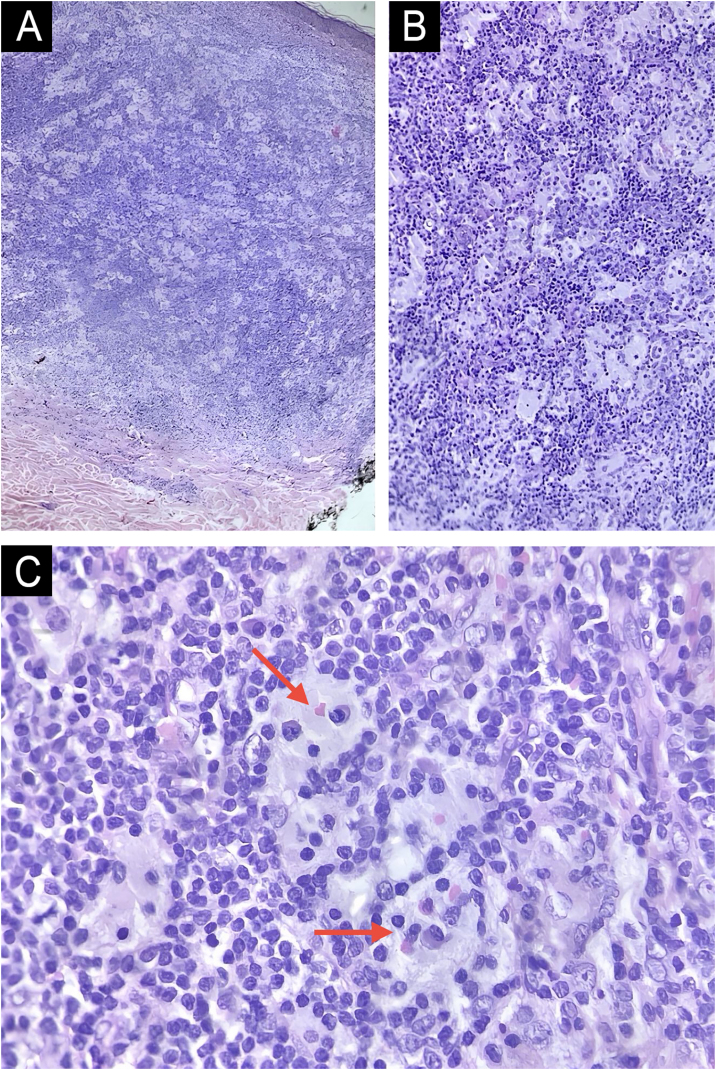
Histopathology of a lesion on the back, stained with Hematoxylin & eosin. (A) Well-defined dermal lymphohistiocytic proliferation without epidermal involvement (Hematoxylin & eosin, ×40). (B) Numerous histiocytes with broad amphophilic cytoplasm interspersed with small lymphocytes (Hematoxylin & eosin, ×100). (C) Presence of emperipolesis demonstrated by the red arrows (Hematoxylin & eosin, ×400).

**Figure 3:**
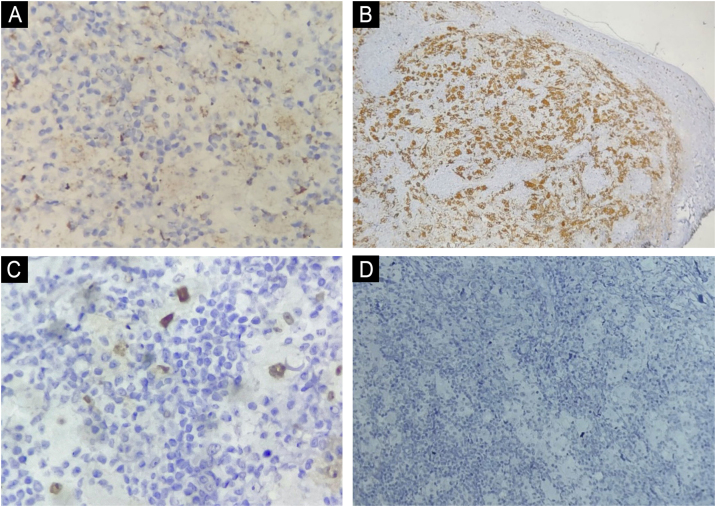
Immunohistochemical analysis. (A) CD68 positive (×40 magnification); (B) S100 positive (×40 magnification); (C) Cyclin D1 positive (×40 magnification); (D) CD1a negative (×10 magnification).

CRDD is a rare condition caused by non-Langerhans cells, described as a distinct clinicopathological entity^1^ and classified in Group C of histiocytoses.^2^ This exclusive skin disease accounts for approximately 3% of all extranodal cases.^3^ Although some studies have attempted to establish a relationship between the disease and viral infections and autoimmune diseases, the etiology remains unknown.^1^, ^4^, ^5^

It primarily affects Caucasian and Asian women, with a mean age of 45 years.^1^, ^2^, ^4^, ^5^ The clinical presentation can vary between papular lesions, plaques, nodules, and asymptomatic, erythematous-brownish tumors of varying sizes.^1^, ^2^, ^3^, ^4^ The most commonly affected sites are the trunk, head, and neck.^1^, ^3^

Histopathology shows dermal lesions and extension into the subcutaneous tissue, with the presence of clusters of histiocytes and emperipolesis, in a mixed inflammatory background with lymphocytes and plasma cells.^4^ Immunohistochemistry is of great importance, especially to exclude differential diagnoses such as Langerhans Cell Histiocytosis.^2^ The main criteria are positivity for S100 and CD68 and negativity for CD1a in histiocytic cells in CRDD.^4^ However, positivity for Cyclin D1 may also be present,^6^, ^7^ as seen in the present case.

Rosai Dorfman-disease is a benign disease with approximately 50% of cases resolving spontaneously.^2^ When exclusively cutaneous involvement occurs, the prognosis becomes even more favorable, as it has a low risk of systemic involvement.^8^ Treatment can include surgical excision, systemic corticosteroids, immunomodulators, chemotherapy, or radiotherapy.^1^ The patient in the present case underwent total excision of the lesions and semiannual follow-up, having already completed two years of clinical follow-up with no recurrences, new lesions, or signs of systemic disease.

## Financial support

None declared.

## Authors' contributions

Carolina Ferreira Baldecerra: Critical review of the literature; drafting and editing of the manuscript; critical review of the manuscript.

Patrícia Imai Zanardi: Critical review of the literature; drafting and editing of the manuscript; critical review of the manuscript.

Rafaella Cristine Zanatta: Critical review of the literature; drafting and editing of the manuscript; critical review of the manuscript.

Silvio César Franco Giovanni Filho: Critical review of the literature; effective participation in research orientation; intellectual participation in the propaedeutic and/or therapeutic conduct of the studied cases; approval of the final version of the manuscript.

Cássio Rafael Moreira: Critical review of the literature; effective participation in research orientation; intellectual participation in the propaedeutic and/or therapeutic conduct of the studied cases; approval of the final version of the manuscript.

## Conflicts of interest

None declared.

## Research data availability

Does not apply.

## Scientific Associate Editor

Hiram Larangeira de Almeida Jr.
